# Effects of low temperature stress on photosynthetic characteristics and antioxidant defense system of flue-cured tobacco

**DOI:** 10.3389/fpls.2025.1638344

**Published:** 2025-09-08

**Authors:** Najam-Ud- Din, Weiguo Ye, Jianjun Chen, Shiyuan Deng, Xianyun Zhong, Yanbing Chen

**Affiliations:** College of Agriculture, South China Agricultural University, Guangzhou, China

**Keywords:** low temperature stress, enzyme activity, physiological effect, photosynthetic characteristics, antioxidant defense

## Abstract

Extreme low-temperature (LT) events, exacerbated by climate change, are increasingly threatening China’s tobacco harvest. This study examines the impact of LT stress on growth biomarkers, antioxidant enzyme activities, and physiological parameters in two tobacco varieties, K326 and Huayan06. Under LT conditions, Huayan06 showed reduced stomatal conductance under LT conditions and a significantly lower PN than K326. Both varieties exhibited an initial increase followed by a decrease in intercellular CO_2_ concentration and chlorophyll fluorescence parameters (Fv/Fm and ΦPSII). Notably, PSII efficiency and photochemical quenching declined sharply when temperatures dropped below 12°C and 16°C. The maximal photochemical efficiency and PN decreased within the first 6–12 hours. K326 leaves displayed higher conductivity and malondialdehyde (MDA) content than Huayan06, indicating more significant LT-induced damage. The ascorbic acid (ASA) and dehydroascorbic acid (DHA) contents varied between the varieties, with Huayan06 showing more resilience. Huayan06 also exhibited a stable glutathione (GSH) content and higher antioxidant enzyme activities superoxide dismutase (SOD), monodehydroascorbate reductase (MDAR), dehydro-ascorbate reductase (DHAR) and ASA during the initial hours of LT exposure, though these declined later. In contrast, K326 showed a substantial rise in DHA and lower GSH levels over time. RuBPase and FDPase activities were significantly reduced under LT treatment. Huayan06 experienced a 44.2% reduction in dry matter accumulation, compared to a 66.4% reduction in K326, demonstrating Huayan06’s superior capacity for dry matter production and resistance to LT stress.

## 1 Introduction

*Nicotiana tabacum*, commonly known as tobacco, is one of the most widely cultivated non-food crops and has been the subject of extensive research (Huang et al., 2021). Given the agricultural significance of tobacco, more than 100 nations produce it for its leaves, primarily used to make cigarettes, cigars, snuff, and other tobacco products (Wu et al., 2020). Environmental temperature, a critical factor influencing the yield and quality of crop plants, affects the distribution, physiological processes, and biochemical activities of these plants (Ahmad et al., 2021). For proper development, tobacco requires an optimal temperature range of 25 to 28°C. Growth ceases at lower temperatures (LT), specifically between 10 to 13°C, and at 1-2°C, seedlings can die (Reichert et al., 2019). Therefore, tobacco is a suitable model plant for studying crops exposed to cold stress. LT affects plant growth, photosynthesis, carbon and nitrogen metabolism, resistance enzyme activity, and dry matter accumulation, eventually leading to growth inhibition and significant production losses (Bhatt et al., 2022). The threat of LT in early spring is a common problem in the tobacco-growing regions of southern China, which poses a serious challenge to producing high-value tobacco leaves (Khalid et al., 2021). While the physiological mechanisms of cold resistance have been extensively studied in crops such as rice, cucumber, pepper, and eggplant, there have been relatively few studies on the physiological and biochemical mechanisms underlying tobacco hardiness (Rani et al., 2020; Zhu et al., 2019).

The capacity of plants to utilize light energy declines sharply under unfavorable conditions, such as cryogenic temperatures, leading to a significant reduction in their PN and potentially exacerbating light inhibition (Ahmad et al., 2022a, 2022b). Studies have shown that under LT conditions, Photosystem I (PSI) is significantly inhibited, decreasing its activity by 70% to 80%. Additionally, the primary light energy transfer efficiency and the potential photosynthetic activity of Photosystem II (PSII) are also inhibited (Kudoh and Sonoike, 2002). When plants experience LT stress, the levels of reactive oxygen species (ROS) increase, leading to the production of MDA, a membrane lipid peroxidation product that compromises membrane integrity (Zhao et al., 2021). Significant findings indicate that the membrane permeability of tobacco seedlings increases as the stress temperature decreases and the duration of stress increases (Li et al., 2020). Membrane lipid peroxidation damages the structure and function of the cytoplasmic membrane, resulting in increased leaf cell membrane permeability, electrolyte leakage, and elevated relative conductivity (Sun et al., 2022). Under normal conditions, plants maintain a scavenging protection system for reactive oxygen species and free radicals, keeping their production and scavenging in a dynamic balance that prevents cellular damage (Kapoor et al., 2019).

Under LT stress, a significant amount of reactive oxygen species (ROS) can be generated in plants, increasing free radical production. This increase results in enhanced membrane lipid peroxidation and the accumulation of peroxidation products (Ouellet et al., 2001). LT stress can also trigger the activation of plant defense systems, which help to mitigate or prevent the damage caused by ROS (Gill and Tuteja, 2010). These defense systems are categorized into enzymatic and non-enzymatic mechanisms. The enzymatic defense system includes enzymes such as SOD, catalase (CAT), peroxidase (POD), ascorbate peroxidase (APX), glutathione reductase (GR), and DHA reductase (DHAR). The non-enzymatic defense system comprises antioxidant substances like GSH, ASA, vitamin E, carotene (CAR), and others (Bhattacharjee, 2005; Wolfe, 1991). The synergistic action of these enzymes and antioxidants effectively removes ROS produced under LT stress, protects membrane integrity, and is a crucial mechanism for enhancing plant cold resistance (Bamagoos et al., 2021).

The adverse effects of early spring LT on flue-cured tobacco are a common challenge in tobacco-growing regions of southern China. LT negatively impacts the growth and development of tobacco seedlings, leading to early flowering, stunted plant growth, a significant reduction in the adequate number of leaves, and decreased tobacco quality (Yang et al., 2019). Despite extensive research, our current understanding of the relationship between the physiological regulation of plant antioxidant metabolism and cold resistance is inconsistent. This inconsistency is primarily due to variations in the genetic background of the plant materials used in different studies, making it difficult to isolate the specific role of the antioxidant metabolic system in cold tolerance. As a result, research findings are often not entirely representative or conclusive.

This study systematically investigated the photosynthetic metabolic characteristics and antioxidant metabolism response mechanisms of two flue-cured tobacco varieties with distinct differences in LT tolerance: K326, an LT-sensitive variety, and HY06, its LT-resistant and early-flowering variant line. This study aimed to elucidate the physiological and biochemical mechanisms underlying cold resistance in flue-cured tobacco, providing a theoretical foundation for improving cold-resistant cultivation practices and breeding efforts.

## 2 Materials and methods

### 2.1 Experimental materials

The tested flue-cured tobacco varieties were (K326), and the new strain Huayan06 (HY06). The experiment was conducted on the campus of South China Agricultural University, Guangzhou, China.

### 2.2 Experimental design

Transferred tobacco materials grown at 22°C to 6°C (In the incubator and the photon flux density and relative humidity remain unchanged) to perform low-temperature stress, measured photosynthetic characteristics and chlorophyll fluorescence-related parameters and antioxidant metabolic defense system-related indicators for 3 replicates at 0, 4, 8, 12, 16, 20, 24 h.

### 2.3 Determination items and methods

#### 2.3.1 Determination of PN and chlorophyll fluorescence parameters

The highest photochemical efficiency of the last full-scale leaf was determined with an FMS-2 portable debug fluorometer (maximal photochemical efficiency, Fv/Fm), Optical system II. Electrons transmit quantum efficiency of photosystem II photochemistry, Φ_PSII_), Photochemical quenching coefficient (photochemical quenching of chlorophyll fluorescence, q_p_), and non-photochemical quenching coefficient (non-photochemical quenching, NPQ). Initial fluorescence yield (Fo) was established by irradiation of the leaf dark adaptation 20 min (<0.05 μmol·m^-2^·s^-1^) before the determination, followed by irradiation of saturated pulsed light (3000 μmol.m^-2^·s^-1^) to determine the highest fluorescence yield (Fm). When the Fm dropped to Fo under the actinic light (180 μmol. m^-2^.s^-1^), and the fluorescence level reached a stable state (fluorescence yield in a steady state of photosynthesis, Fs), the samples were irradiated with saturated pulsed light to measure the maximum fluorescence under light (maximal fluorescence yield at actinic light, Fm’). Finally, the actinic lights were turned off, and samples with far-red were irradiated r-red light, and the assay was repeated four times for each treatment. According to (Castro-Arevalo et al., 2008), PSII is calculated. Maximum photochemical efficiency (F_v_/F_m_) = (F_m_-F_o_)/Fm under dark adaptation; photosystem II. Photosynthetic electron transport quantum efficiency (ΦPSII.) = (Fm’-Fs)/Fm’; antenna conversion efficiency (Fv’/Fm’) = (Fm’-Fo’)/Fm’;P SII activity (Fv/Fo); photochemical extinction coefficient (qP) = (Fm’-Fs)/(Fm’-Fo’).

#### 2.3.2 Determination of MDA content

MDA content was determined, followed by (Li et al., 2022). 0.5 g of clean leaves were mixed with 5 mL of 0.1% mL trichloroacetic acid (TCA) and then centrifuged at 8000 rpm at 4°C for 15 min. 1.5 mL of this supernatant solution and 2.5 ml of 50% thiobarbituric acid (TBA) solution were mixed evenly and reacted in water bath for 20 min. The samples were cooled rapidly in an ice bath, and the final mixture was centrifuged at 8000 rpm at 4°C for 15 min. We measured the absorbance of the supernatants at 532 nm, 600 nm, and 450 nm. The levels of MDA were calculated with a 155 mM^-1^ cm^-1^ extinction coefficient.

#### 2.3.3 Determination of ASA, DHA

The ASA and DHA content were determined according to the method of (Zhai et al., 2023) with possible adjustments. Briefly, a total of 0.5 g of fresh tobacco leaves were homogenized with 2.5 mL of precooled 5% sulfosalicylic acid and a little quartz sand. The mixture was grounded in an ice bath and transferred into a centrifuge tube. After that, added 24 μL of 1.84 mol·L-1 triethanolamine to 100 μL of supernatant to neutralize the sample solution. Then, added 250 μL of 50 mmol·L-1 PBS (pH 7.5) containing 2.5 mmol·L-1 EDTA and 50 μL of 10 mmol·L^-1^ DTT (dithiothreitol). The solution was kept at 25°C for 10 minutes to reduce DHA to ASA. Finally, we added 50 μL of 0.5% ethyl maleimide and mixed well to eradicate the remaining DTT. Mix well, water bath at 40°C for 1 h, and measure the OD value at 525 nm. This method examines the amount of total ASA in the sample. In determining ASA, distilled water of equal volume can replace the above DTT and ethyl maleimide. Applying 5% sulfosalicylic acid as solvent created the ASA standard curve using the same procedure.

#### 2.3.4 Determination of GSH, GSSG

A 50 μL supernatant solution was obtained and diluted to 100% with 5% sulfosalicylic acid (i.e., by adding 50 μL of 5% sulfosalicylic acid). Then, 24 μL of 1.84 mol·L^-1^ triethanolamine was added to neutralize the sample solution. Then, 50 μL of 10% vinyl pyridine (prepared with 70% ethanol) was added, and the mixture was incubated in a 25°C-water bath for 1 hour to eliminate GSH. Subsequently, 706 μL of 50 mol·L^-1^ pH 7.5 PBS was added, containing 2.5 mmol·L^-1^ EDTA. It was followed by the addition of 20 μL of 10 mmol·L^-1^ NADPH and 80 μL of 12.5 mmol·L^-1^ DTNB (disulfide nitrobenzoic acid), and the mixture was thoroughly mixed and kept at 25°C for 10 minutes. Then, 20 μL of 50 U/mL GR was added, making the total volume 1 mL. The mixture was immediately mixed well, and the OD value was measured at 3 minutes. To investigate total (GSH + GSSG), vinyl pyridine was replaced with an equal volume of distilled water. Using 5% sulfosalicylic acid as the solvent, the GSSG standard curve was created using the same method (Cui et al., 2015).

#### 2.3.5 Determination of SOD activity

The SOD activity was evaluated, following the procedure of (Zhang et al., 2023), with minor modifications. Briefly. 0.5 g clean leaves were applied and crushed with 5 mL of cold (4°C) 0.05 mol. L^-1^ PBS with pH 7.8 containing 1% polyvinylpolypyrrolidone (PVPP) and a modest quantity of quartz sand ice bath. All mixture was centrifuged at 8000 rpm for 15 min at 4 °C. The supernatant was the enzyme solution. 3 mL of the reaction solution contains 1.5 mL of 0.05 mol·L^-1^ PBS with pH 7.8, 0.3 mL of 130 mmol.L^-1^ methionine, 0.3 mL of 0.75 mmol.L^-1^ Nitro tetrazolium chloride (NBT), 0.3 mL of 0.1 mmol.L^-1^ disodium EDTA, 0.3 mL of 0.02 mmol.L^-1^ riboflavin, 0.05 mL of the enzyme solution and 0.25 mL of distilled water. A phosphate buffer solution as the control replaced the enzyme solution. After mixing, the reaction was conducted under 4000 lx light for 20 min, then carried out under no light. Blank, the absorbance values at 560 nm wavelength were measured, and the inhibition of NBT photoreduction by 50% was used to indicate an enzyme activity.

#### 2.3.6 Determination of peroxidase activity

Peroxidase activity was determined following the method of (Hafeez et al., 2022) with minor modifications. Briefly, 0.5 g of clean leaf tissue was homogenized with 5 mL of 0.05 mol·L^-1^ phosphate-buffered saline (PBS, pH 7.8) containing 1% (w/v) (PVPP) and a small amount of quartz sand in an ice bath. The homogenate was centrifuged at 8000 rpm for 15 minutes at 4°C, and the supernatant was collected as the crude enzyme extract. For the assay, a 3 mL reaction mixture was prepared containing 2 mL of 0.3% H_2_O_2_, 0.95 mL of 0.2% guaiacol, and 1 mL of PBS (pH 7.0). The reaction was initiated by adding 0.05 mL of the crude enzyme extract, and the change in absorbance at 470 nm was recorded. One unit of peroxidase activity was defined as a 0.01 increase in OD per minute.

### 2.4 Determination of catalase activity

Catalase activity was determined according to the method of (Anderson, 2002) with slight modifications. Briefly, 2.5 g of clean leaf tissue was homogenized in an ice bath with 5 mL of 50 mmol·L^-1^ phosphate buffer solution (pH 7.8, at 4°C), 20 mmol·L^-1^ H_2_O_2_, and a small amount of quartz sand. The homogenates were then centrifuged at 4000 rpm for 10 minutes at 4°C. The absorbance of the resulting supernatant was measured at 240 nm to assess catalase activity.

### 2.5 Activity determination of key enzymes APX, MDAR, DHAR, and GR in the ASA-GSH pathway

According to the method of (Yue-Hua et al., 2003), the enzyme solution was extracted with 0.5 g cleaned leaves and crushed with liquid nitrogen in a mortar. After thawing, we added 3.5 mL PBS (containing 2% PVPP, 1 mmol·L^-1^ EDTA (sodium salt, fresh preparation), 1 mmol. L^-1^ ASA, and 0.25% Triton X-100, pH 7.6). Then, we added 1.5 mL of saturated ammonium sulfate solution and stirred the mixture thoroughly. We filtered the solution with 4 layers of gauze. All the materials were centrifuged at 16000 rpm with a temperature of 4°C for 20 min. The supernatant was the crude enzyme extract.

APX activity determination:

To determine the APX), the method of (Wu et al., 2015) was followed. Briefly, 0.3 g of fresh tobacco leaves were homogenized in 5 ml of 50 mM cold phosphate buffer (pH 7.0) with 1 mM ethylenediaminetetraacetic acid (EDTA) and 1% (w/v) (PVP). 1 mM ASA was then added. The resulting mixtures were centrifuged for 20 minutes at 4°C at 12,000 × g, and the enzyme activity assays were performed using the supernatants.

Determination of MDAR activity:

The MDAR activity was determined according to the method described by (Tanaka et al., 2021), with slight modifications. Briefly, a total of 0.2 grams of tobacco leaves, previously frozen in liquid nitrogen, were ground and homogenized in 1 mL of 50 mM MES/KOH buffer (pH 6.0) containing 1 mM ascorbate (ASC), 40 mM KCl, and 2 mM CaCl_2_. The homogenates were centrifuged at 15,300×g for 15 minutes at 4°C, and the resulting supernatant was immediately used for enzyme assays. For the assay, 50 µL of the enzyme extract was added to 940 µL of a reaction mixture consisting of 0.2 mM NADH or NADPH, 2.5 mM ASC, and 50 mM HEPES buffer (pH 7.6). The reaction was initiated by the addition of 5 µL (0.2 units) of ascorbate oxidase. Enzyme activity was measured by monitoring the decrease in absorbance at 340 nm using an extinction coefficient of 6.2 mM^-1^ cm^-1^.

The GR activity was determined following the method of (Kusvuran, 2021), with slight modifications. The reaction mixture consisted of 0.86 mL of 1 mM GSSG, 0.1 mL of 2 mM NADPH prepared in phosphate buffer solution (pH 7.6), and 0.04 mL of enzyme extract. The change in optical density (OD) was recorded at 340 nm after 1 minute of reaction.

### 2.6 Determination of photosynthetic metabolic enzymes

#### 2.6.1 RuBPCase activity assay

The RuBPCase activity was investigated according to the method of (Hu et al., 2012) with possible adjustments. Briefly, 10 g of green leaves were cleaned and dehydrated. Next, added 10 mL of pre-cooled extraction medium and filtered the mixture. The resulting high-speed homogenate was filtered through 4 layers of gauze and then centrifuged at 20,000 rpm with a temperature of 4°C for 15 minutes. The supernatant was used to examine the enzyme activity. The prepared reaction system (0.2 mL of 5 mmol·L^-1^ NADH, 0.02 mL of 50 mmol·L^-1^ ATP, 0.2 mL of 50 mmol·L^-1^ creatine phosphate, 0.2 mL of 0.2 mol·L^-1^ NaHCO_3_, 0.1 mL of 160 μmol·L^-1^ phosphocreatine kinase, 0.1 mL of 160 μmol·L^-1^ phosphoglycerate kinase, and 0.1 mL of 160 μmol·L^-1^ glyceraldehyde-3-phosphate dehydrogenase) was shaken and poured into the cuvette. Distilled water was used as a blank, and the optical density of the reaction system at 340 nm was measured as the zero value. Then, 0.1 mL of RuBP was added to the cuvette, and the time was recorded immediately. The absorbance was measured every 30 seconds for a total of 3 minutes. The enzyme activity was determined by the absolute value of the absorbance decrease from zero to the first minute.

#### 2.6.2 Determination of fructose-1,6-diphosphate esterase activity

The FDPase activity was determined by following the method of (Nah et al., 2001) with slight modifications. Briefly, 5 g of chopped tobacco leaves were inserted into a mortar, and 20 mL of separation medium (0.35 M sucrose, containing 0.05 M Tris-HCl, 5 mM MgCl_2_, 5 mM L-cysteine, 2 mM EDTA Na, pH 7.5) were added. The suspension was sorted through 4 layers of 100 mesh nylon cloth. The supernatant was centrifuged for 3min at 4000 rpm. After centrifugation for 90 s, the precipitate was chloroplast. Suspend with 2 mL medium (wash once), then 4500 rpm centrifuged for 2 min. The washed chloroplasts were immediately suspended with 2 mL of distilled water for 15 min to break the chloroplasts, which contained FDP esterase.

The reaction solution was prepared with the following components: 100 μM Tris-HCl (pH 8.0), 5 μM GSH, 0.1 μM EDTA-Na, and 15 μM MgCl_2_. Then, 0.1 mL of enzyme solution and 1.3 mL of the reaction solution were added, and the mixture was kept at a constant temperature of 30°C for about 5 minutes. Following this, 0.1 mL of 5.0 μM FDP was added. The initial reaction of MFDP sodium salt was carried out at 30°C for 10 minutes. To conclude the reaction, 0.5 mL of 12% trichloroacetic acid was added. The inorganic phosphorus was then determined using the molybdenum blue method.

## 3 Data processing and analysis

The initial experimental data were processed in Excel and analyzed by DPS 7.05, and all the graphs were developed by Sigmaplot10.0 software.

## 4 Results

### 4.1 Impacts of different LT treatments on photosynthetic characteristics and chlorophyll fluorescence characteristics of flue-cured tobacco

#### 4.1.1 Effect on photosynthetic characteristics of flue-cured tobacco

**Figure 1:**
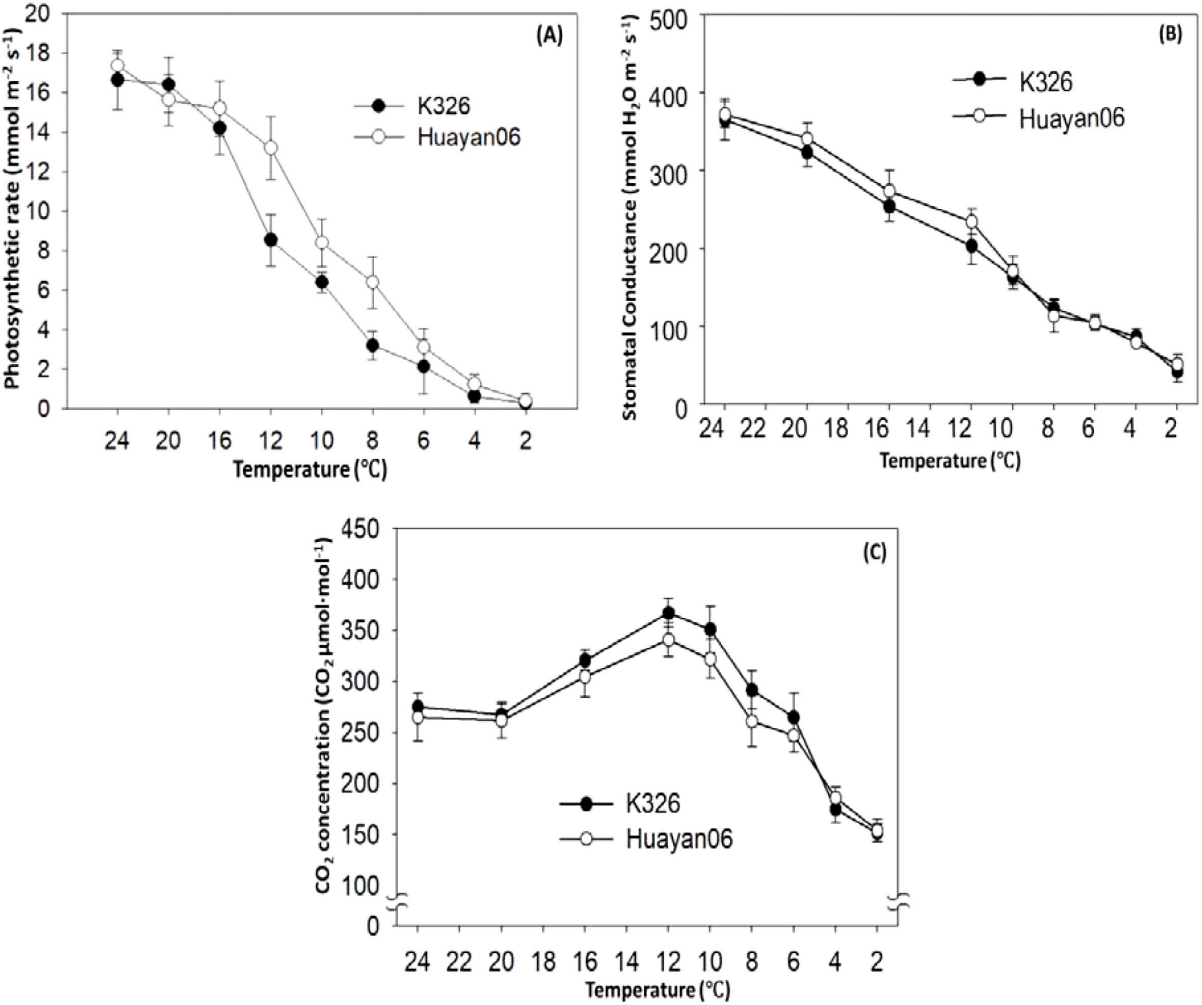
Changes of PN **(A)**, stomatal conductance **(B)**, and intercellular CO_2_ concentration **(C)** of flue-cured tobacco leaves at various temperatures.

**Figure 1** showed the variations in PN (a), stomatal conductance (b), and intercellular F (c) of the 8th fully opened leaf of flue-cured tobacco after 6 h treatment at different temperatures. The photosynthetic frequency of flue-cured tobacco leaves decreased with the treatment temperature. When the temperature dropped from 24°C to below 16°C, the PN of flue-cured tobacco leaves decreased significantly, and there were significant differences among the varieties. The PN of the Huayan 06 strain was considerably shorter than that of the K326 species. Stomatal conductance also decreased with the temperature decrease, but there was no significant difference among varieties. However, the change in intercellular CO_2_ concentration in leaves demonstrated a tendency to rise and decrease with the reduction in treatment temperature.

#### 4.1.2 Effect on chlorophyll fluorescence parameters of Flue-cured Tobacco

**Figure 2:**
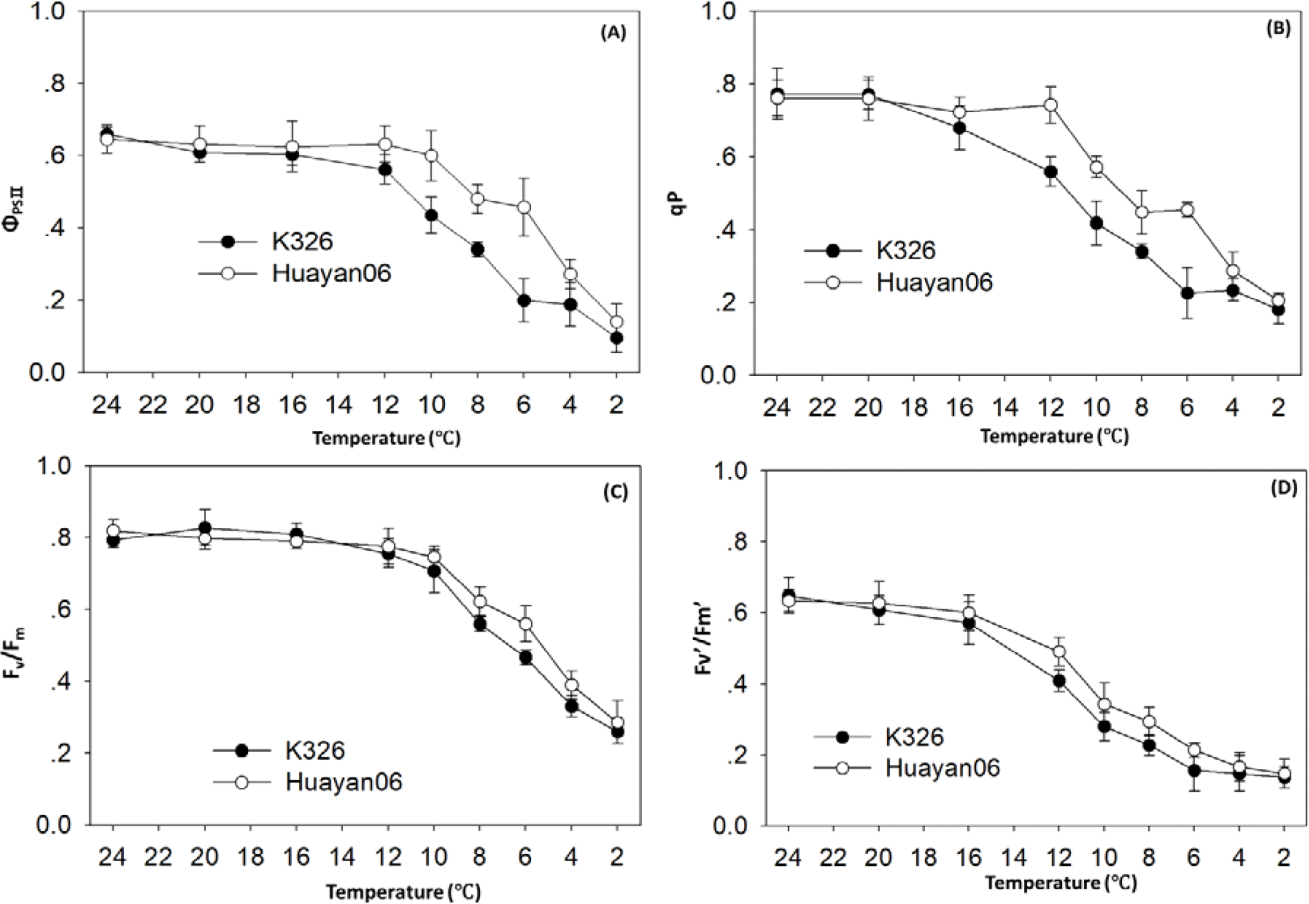
Chlorophyll fluorescence parameters of Flue-cured Tobacco Leaves under distinct temperature treatments Φ Changes of Φ_PSII_ **(A)**, qP **(B)**, F_v_/F_m_ **(C)**, Fv’/Fm’ **(D)**.

Chlorophyll fluorescence factors of the 8th fully opened leaf of flue-cured tobacco treated at several temperatures for 6 h. The changes of Φ_PSII、_qP_、_F_v_/F_m_ and Fv’/Fm’ are shown in **Figures 2A–D**.

Φ_PSII_ is the quantum effectiveness of photosynthetic electron transfer of PSII, which reveals the efficiency of the photoelectron absorbed by plants to supply PSII reaction center. It can be found from **Figure 2A** that when the treatment temperature dropped from 24°C to 16°C, Φ_PSII_ had no obvious change. When the temperature dropped below 12°C, Φ_PSII_ decreased significantly, and the difference between varieties were significant. The decrease of Huayan 06 was significantly less than that of K326.

QP is the photochemical quenching coefficient, which reflects the part of light energy absorbed by PSII antenna pigment used for photochemical electron transfer. The higher the photochemical quenching coefficient QP, the superior the electron transfer activity of PSII (Liu K. et al., 2022). It can be observed from **Figure 2B** that as the treatment temperature decreases from 24°C to 20°C, the photochemical quenching coefficient QP of flue-cured tobacco leaves did not change significantly. However, when the temperature drops below 16°C, the photochemical quenching coefficient QP of flue-cured tobacco leaves decreases significantly, and the difference between varieties were noticeable. The reduction range of Huayan 06 was significantly less than that of K326. Above, the changes of Φ_PSII_ and QP showed that the electron transfer activity of PSII in Huayan 06 leaves were relatively high compared with K326 at a lower temperature.

F_v_’/F_m_’ is the maximum photochemical quantum yield of PS II, which reflects the intrinsic light energy conversion efficiency of PSII reaction center or the maximum light energy conversion efficiency of PSII. It was measured after the dark adaptation of leaves for 20 min. It can be seen from **Figure 2C** that when the treatment temperature was reduced from 24°C to 12°C, F_v_’/F_m_’ had no noticeable change, but when the temperature was reduced below 12°C, F_v_’/F_m_’ decreased significantly, and the decline of K326 strain was slightly greater than that of Huayan 06.

F_v_’/F_m_’ is the effective photochemical quantum yield of PS II, which reflects the primary light energy capture efficiency of the PSII reaction center. The leaves were directly measured under light without dark adaptation. It can be seen from **Figure 2D** that F_v_’/F_m_’ was affected by temperature treatment, and the trend of F_v_’/F_m_’ performance was the same. When the temperature dropped below 12°C, F_v_’/decreased significantly, and the decline of the K326 strain was slightly greater than that of Huayan 06. This also showed that the light energy gain efficiency and light energy conversion efficiency of Huayan 06 were higher than those of K326 at LT.

### 4.2 Impacts of LT treatment times on flue-cured tobacco’s photosynthetic and chlorophyll fluorescence characteristics

#### 4.2.1 Effects on photosynthetic rate, stomatal conductance, and intercellular CO_2_ concentration of Flue-cured Tobacco

**Figure 3:**
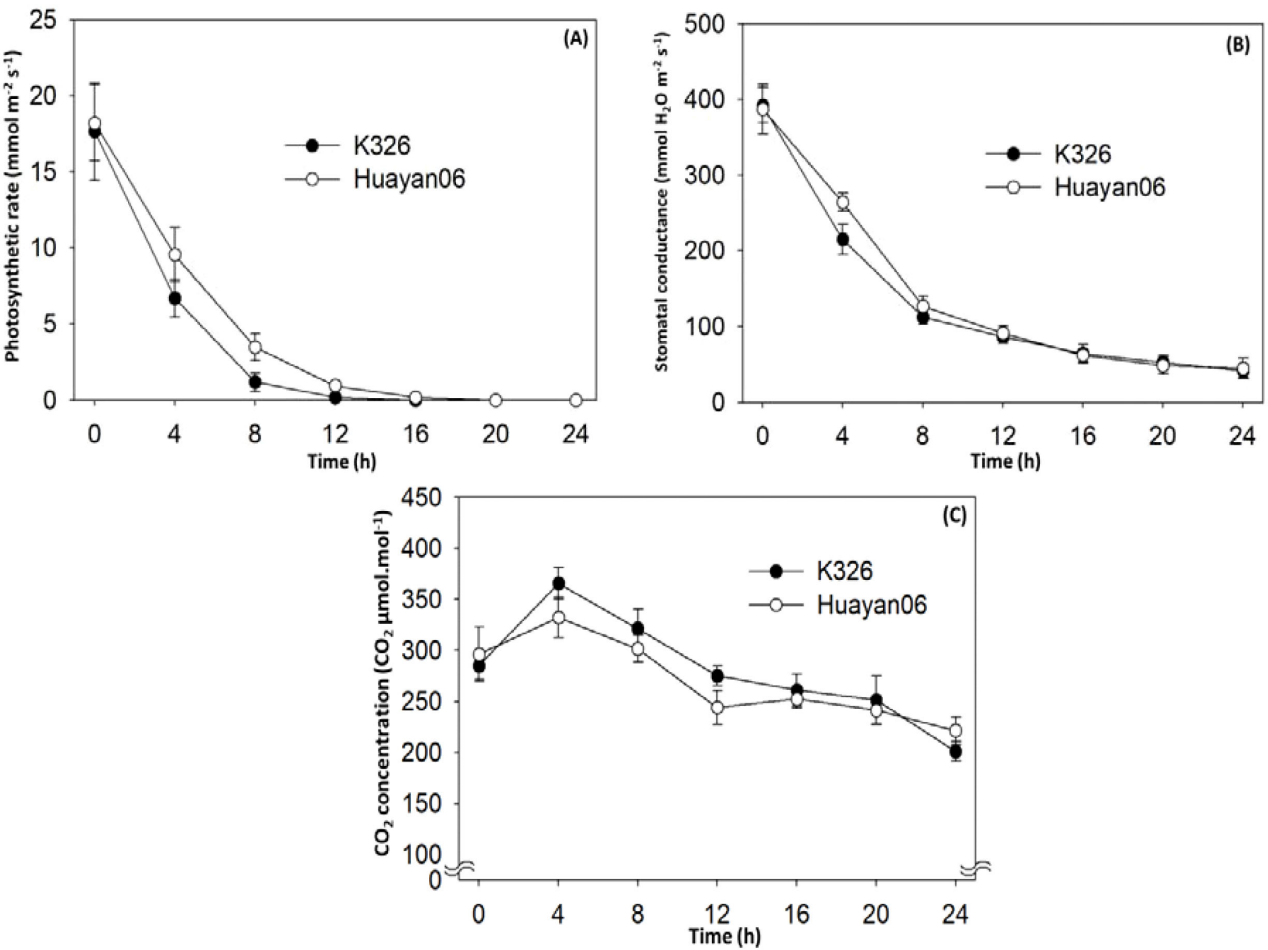
Effect of LT (6°C) treatment time on PN **(A)**, stomatal conductance **(B)** and intercellular CO_2_ concentration **(C)**.

**Figure 3** shows the changes in photosynthetic characteristic parameters of flue-cured tobacco leaves treated at 6°C numerous times. It can be seen from **Figure 3** that the photosynthetic characteristics of the leaves of the two flue-cured tobacco variations tested were appreciably affected by the LT treatment time. With the expansion of treatment time, leaves’ PN and stomatal conductance decreased sharply. At 8 h after treatment, leaves’ PN and stomatal conductance decreased very low. At 16 h after treatment, the PN of leaves were almost reduced to 0, and the leaves did not undergo photosynthesis. The intercellular CO_2_ concentration increased significantly within 0~4 h after LT treatment and then diminished with the extension of the treatment period. There were significant differences among varieties during 0~12 h of LT treatment, and the change range of intercellular CO_2_ concentration in K326 leaves were greater than that in Huayan 06 leaves.

#### 4.2.2 Chlorophyll fluorescence parameters (F_v_/F_m_、Φ_PSII_)) impact

**Figure 4:**
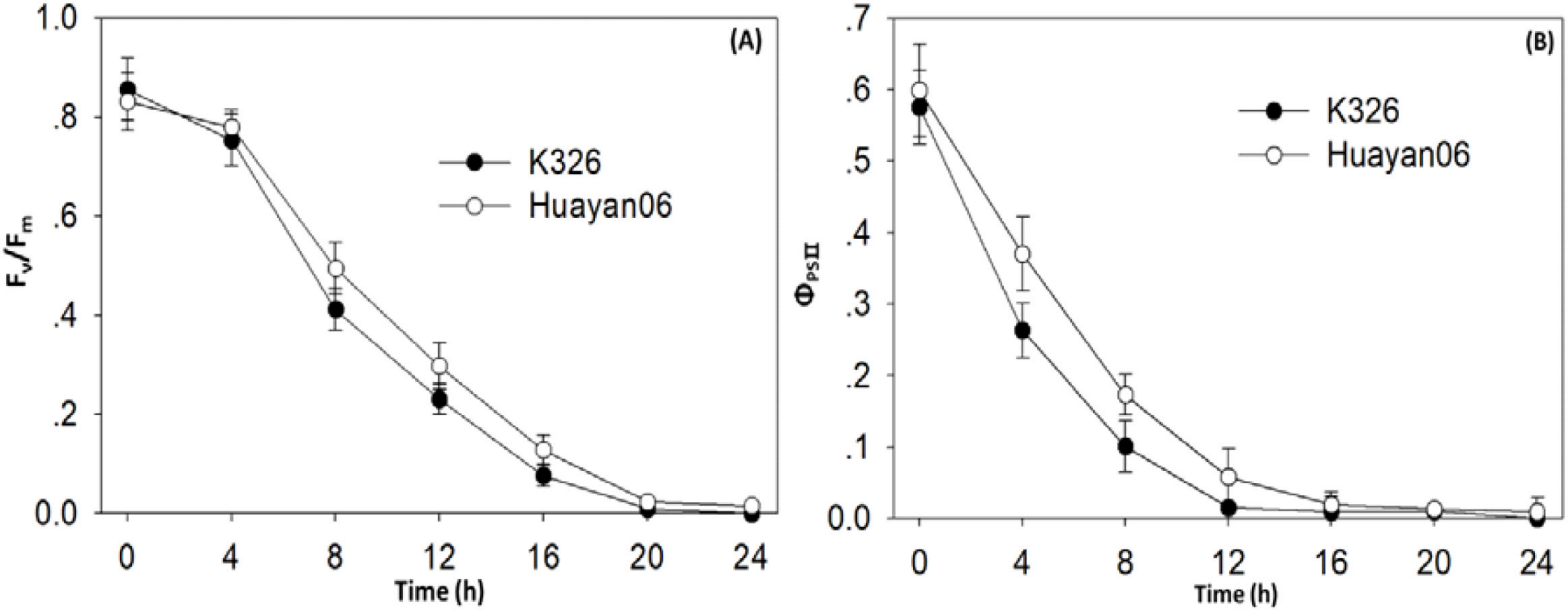
Effects of LT treatment time on chlorophyll fluorescence parameters impact F_v_/F_m_ **(A)** and Φ_PSII_ **(B)**.

As can be seen from **Figure 4**, chlorophyll fluorescence parameters Fv/Fm and Φ_PSII_ were also extensively influenced by LT treatment time. With the extension of treatment time, Fv/Fm and Φ_PSII_ decreased significantly, and there was a significant difference among varieties 1–6 h after treatment. The Fv/Fm and the decrease of Φ_PSII_ were significantly greater than that of Huayan06.

### 4.3 Effect of variable temperature treatment on PN and maximum photochemical efficiency (fv/fm) of Flue-cured tobacco

**Figure 5:**
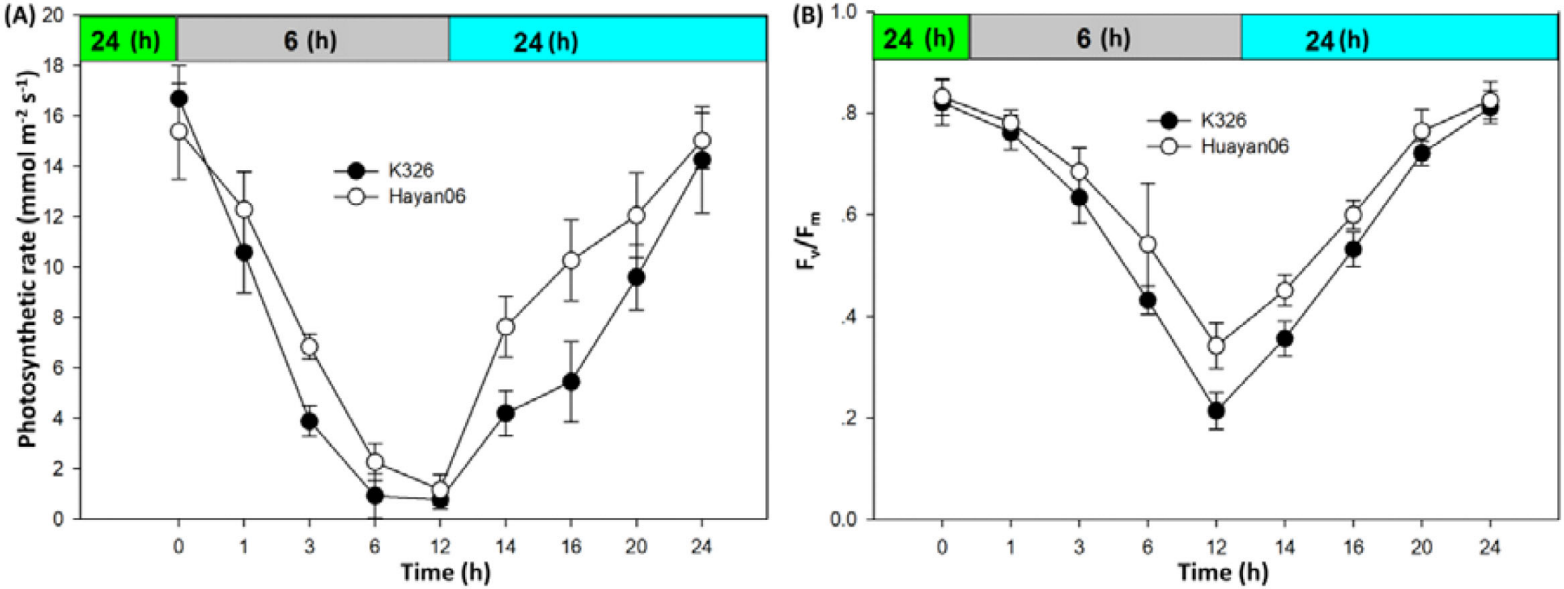
Changes of PN **(A)** and maximum photochemical efficiency **(B)** of Flue-cured Tobacco Leaves under variable temperature treatment.

**Figure 5** shows the changes in PN and maximum photochemical efficiency of leaves of two varieties under variable temperature treatment within 24 h. The trend of PN and maximum photochemical efficiency was the same under the influence of temperature change. They decreased sharply in the first 6 h of LT treatment at 6°C. With the treatment time extending to 12 h, the PN and maximum photochemical efficiency reduced further, especially the maximum photochemical efficiency. After LT treatment for 12 h, the PN and maximum photochemical efficiency of flue-cured tobacco leaves can be restored to that before LT treatment, which indicates the effects of short-term (12 h) LT treatment on photosynthesis and chlorophyll fluorescence of flue-cured tobacco leaves can be restored.

### 4.4 Response of antioxidant metabolic defense system of flue-cured tobacco leaves to LT stress

#### 4.4.1 Effect of LT on electrical conductivity and MDA content of flue-cured tobacco leaves

**Figure 6:**
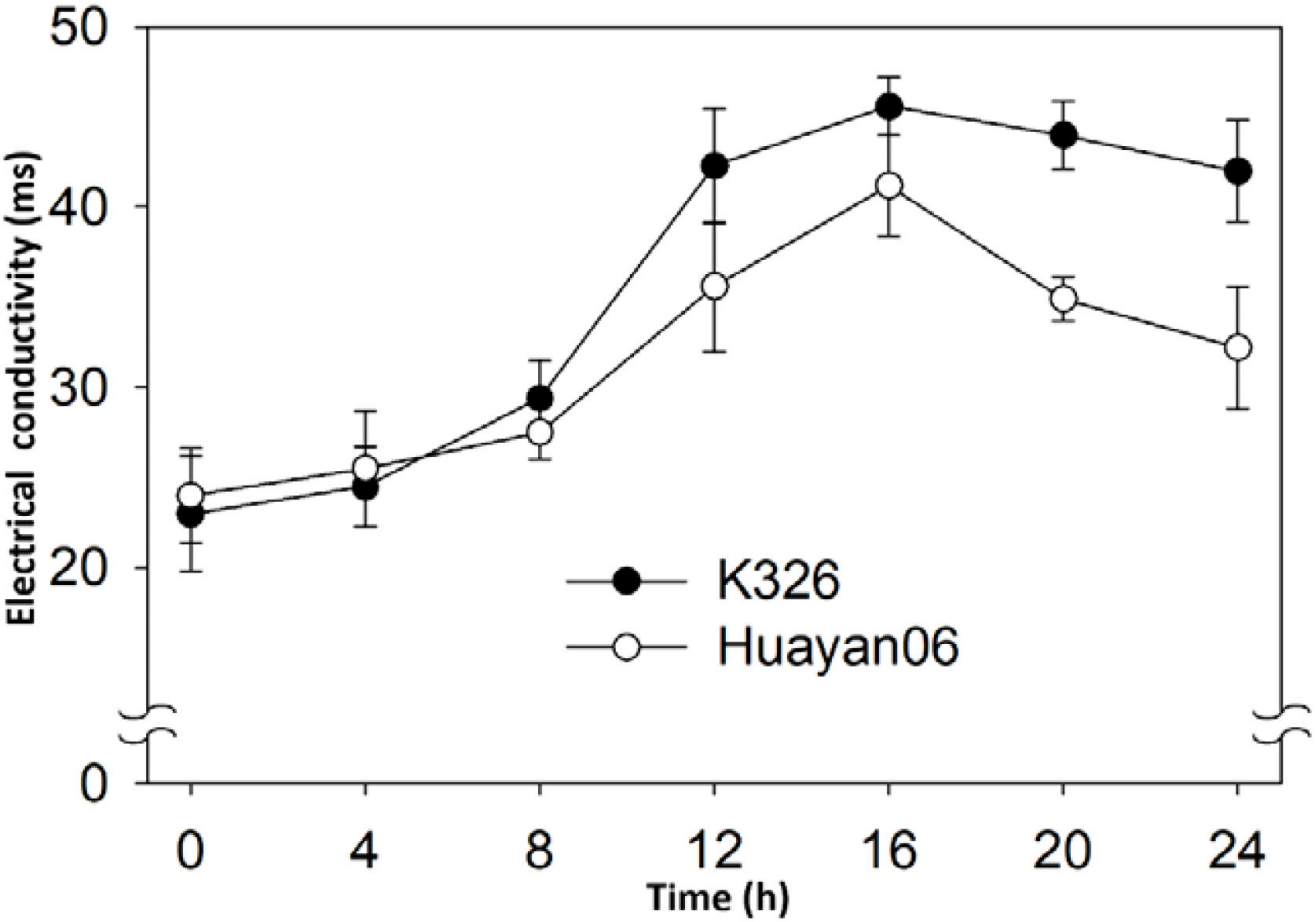
Effect of LT treatment time on the conductivity of flue-cured tobacco leaves.

The change of conductivity of flue-cured tobacco leaves within 24 h after LT treatment at 6 °C is shown in **Figure 6**. The conductivity of leaves increased within 0~8 h after treatment, but there was no significant difference with that before LT treatment. When the LT treatment time was extended to 12 h, the conductivity of flue-cured tobacco leaves increased significantly until the conductivity of flue-cured tobacco leaves increased to the peak at 16 h after treatment. Then, with the extension of treatment time, the conductivity of flue-cured tobacco leaves began to decrease but remained at a higher level than before treatment. It can also be seen from **Figure 6** that there were obvious differences among varieties in the change of conductivity of flue-cured tobacco leaves after LT treatment. During the 8~24 h period of LT treatment, the conductivity of K326 leaves were considerably greater than that of Huayan06, which reveals that the damage of K326 leaves after LT treatment is more significant than that of Huayan 06. On the contrary, it also indicates that the resistance of Huayan 06 leaves to LT was more vital than that of K326.

**Figure 7:**
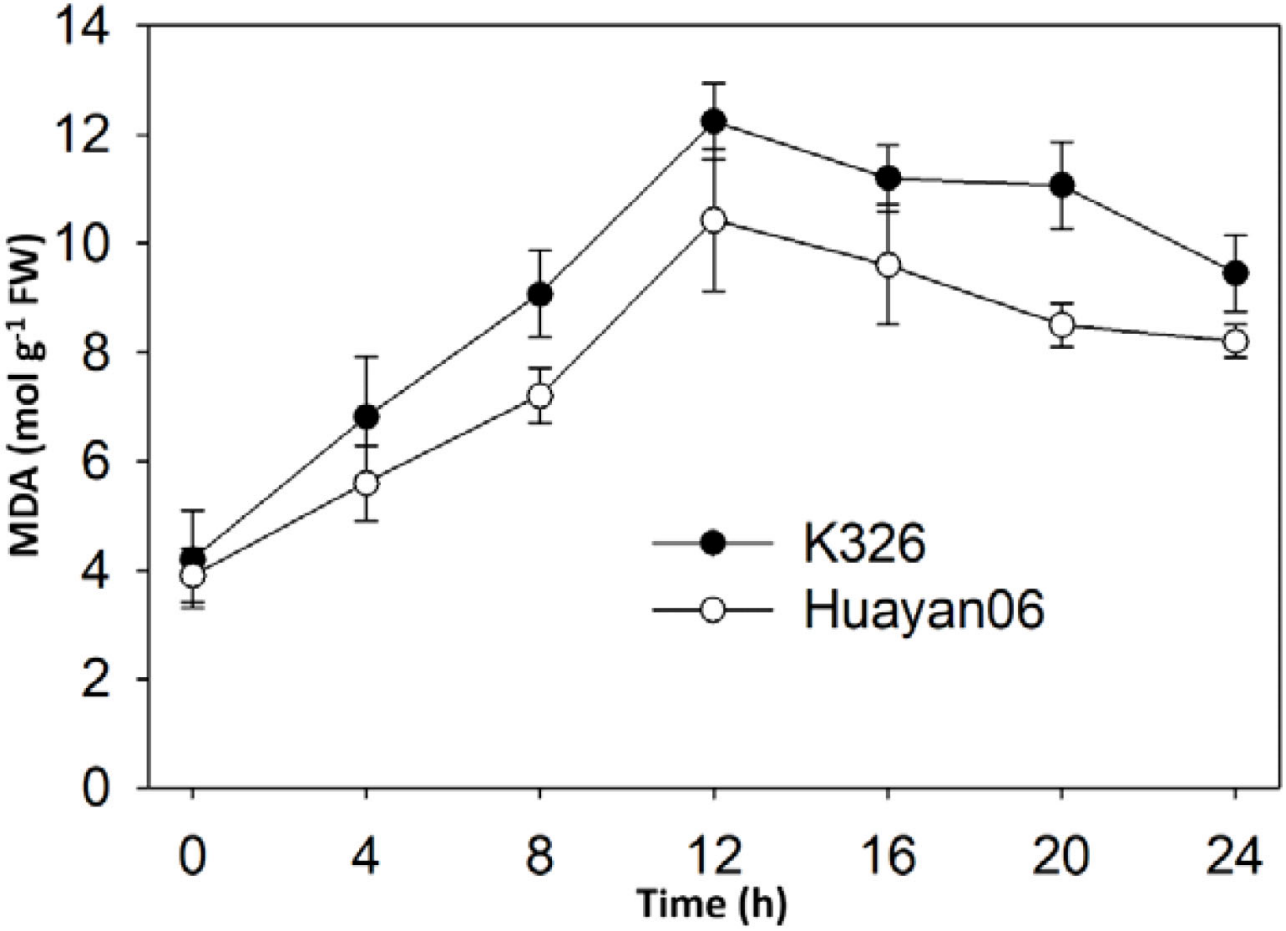
Effect of LT treatment time on MDA content in flue-cured tobacco leaves.

**Figure 7** displays the change in MDA content of membrane lipid peroxidation products in flue-cured tobacco leaves within 24 h after LT treatment. The MDA content in leaves improved significantly after LT treatment. At 12h after treatment, the MDA content in leaves peaked. Then, with the extension of treatment time, the MDA content in leaves decreased slightly but persisted at a high level. At 24 h after LT treatment, the MDA content in leaves was significantly higher than before treatment, and there was no significant difference in MDA content between the untreated and the treated leaves during 8~12 h. It can also be seen from **Figure 7** that the change in MDA content also varies with flue-cured tobacco varieties. Within 24 h after LT treatment, the MDA content in the leaves of Huayan 06 was significantly lower than that of K326, consistent with the change of leaf conductivity above.

#### 4.4.2 Effects of non-enzymatic protective substances in flue-cured tobacco leaves under LT stress

**Figure 8:**
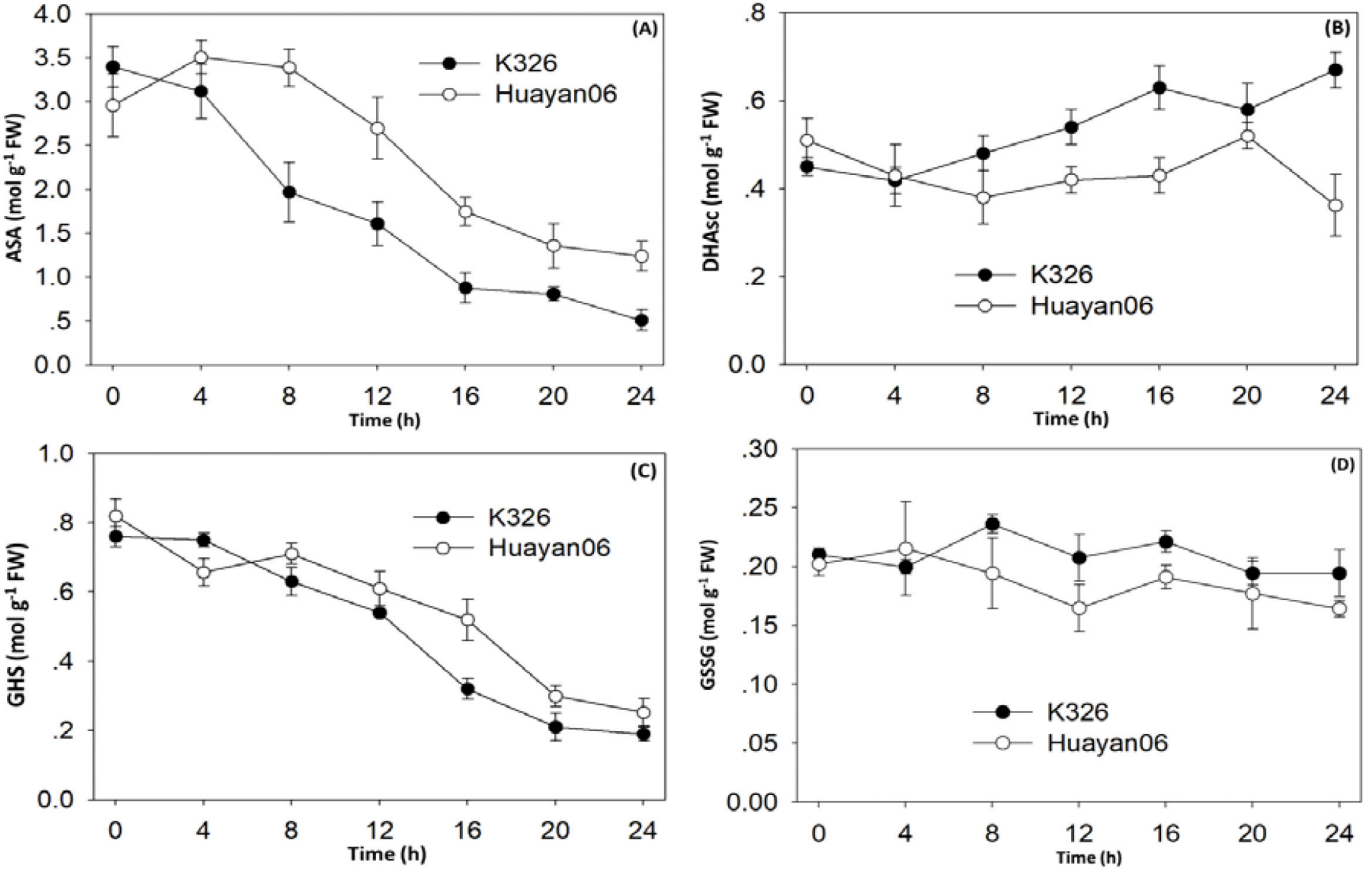
Changes in the content of non-enzymatic protective substances **(A)** AsA, **(B)** DHA, **(C)** GSH & **(D)** GSSG in flue-cured tobacco leaves K326 under different durations of LT treatment.

**Figure 8A** shows the changes in ASA, DHA, reduced GSH, and oxidized glutathione (GSSG). Within 4 h after LT treatment, there was no major change between ASA and that before treatment. Still, with the extension of treatment time, the content of ASA in leaves of K326 decreased sharply after 4–16 h treatment. In contrast, Huayan 06 significantly reduced after 8h treatment, but the decrease was smaller than that of K326 (**Figure 8B**). The changing trend of DHA at different times after LT treatment was opposite to that of ASA, and there were significant differences among varieties. Within 0–4 h after LT treatment, the changes of DHA in the leaves of the two tested types were not noticeable and decreased slightly. However, at 4–20 h after treatment, the content of DHA in the leaves of Huayan 06 showed decreasing trend followed by an increase, while the range of DHA in the leaves of K326 showed a significant upward trend. It can be seen from **Figure 8C** that the changing trend of GSH content in leaves of two flue-cured tobacco varieties at different periods after LT treatment was the same.

With the extension of treatment time, the GSH content in leaves gradually decreased. At 4 h after treatment, the GSH content in the leaves of Huayan 06 decreased, which was significantly lower than that of K326. At 8–24 h after treatment, the GSH content in leaves of Huayan 06 was higher than that of K326. The content of oxidized glutathione (GSSG) (see **Figure 8D**) changed slightly in different periods of LT treatment, and the content of GSSG in leaves of the two varieties remained high ranking during the whole treatment period.

#### 4.4.3 Responses of antioxidant protective enzymes SOD, POD, CAT, and APX to LT stress

**Figure 9:**
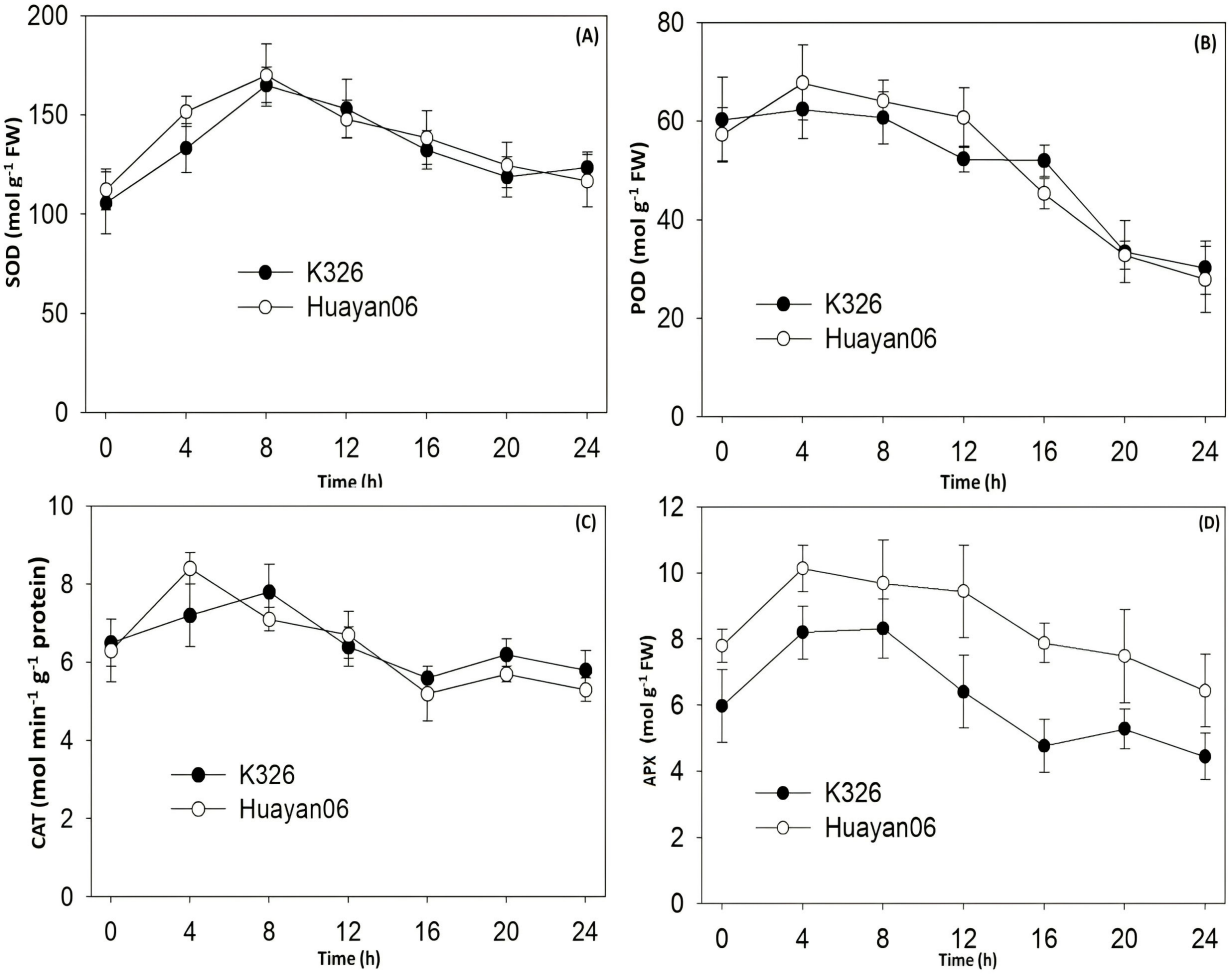
Response of LT treatment on flue-cured tobacco leaves antioxidant enzyme activity (**(A)** SOD, **(B)** POD, **(C)** CAT, **(D)** APX) under different time duration.

**Figure 9A** shows the differences in the activities of antioxidant enzymes SOD, POD, CAT, and APX in flue-cured tobacco leaves at different periods of LT treatment. It can be seen from **Figure 9B** that the SOD activity increased significantly at 0–4 h after LT treatment and then reduced progressively with the extension of treatment time (8–20 h after treatment) and recovered to the level before treatment at 20–24 h after treatment.

The activity changes of POD, CAT, and APX were similar to those of SOD. POD, CAT, and APX activity increased at 0–8 h after LT treatment. Still, it reduced steadily with the extension of treatment time, especially the activity of POD, which was significantly reduced after 24 h of treatment (**Figure 9C**). However, there was no substantial change in the activities of SOD, POD, and CAT between the two varieties (strains) during the whole LT treatment time. However, the APX activity of Huayan 06 was significantly higher than that of K326, indicating that the increase of APX activity in Huayan 06 under LT stress was a primary reason for its strong ability to scavenge reactive oxygen species (**Figure 9D**).

#### 4.4.4 Responses of antioxidant regenerating enzymes MDAR, DHAR, and GR to LT stress

**Figure 10:**
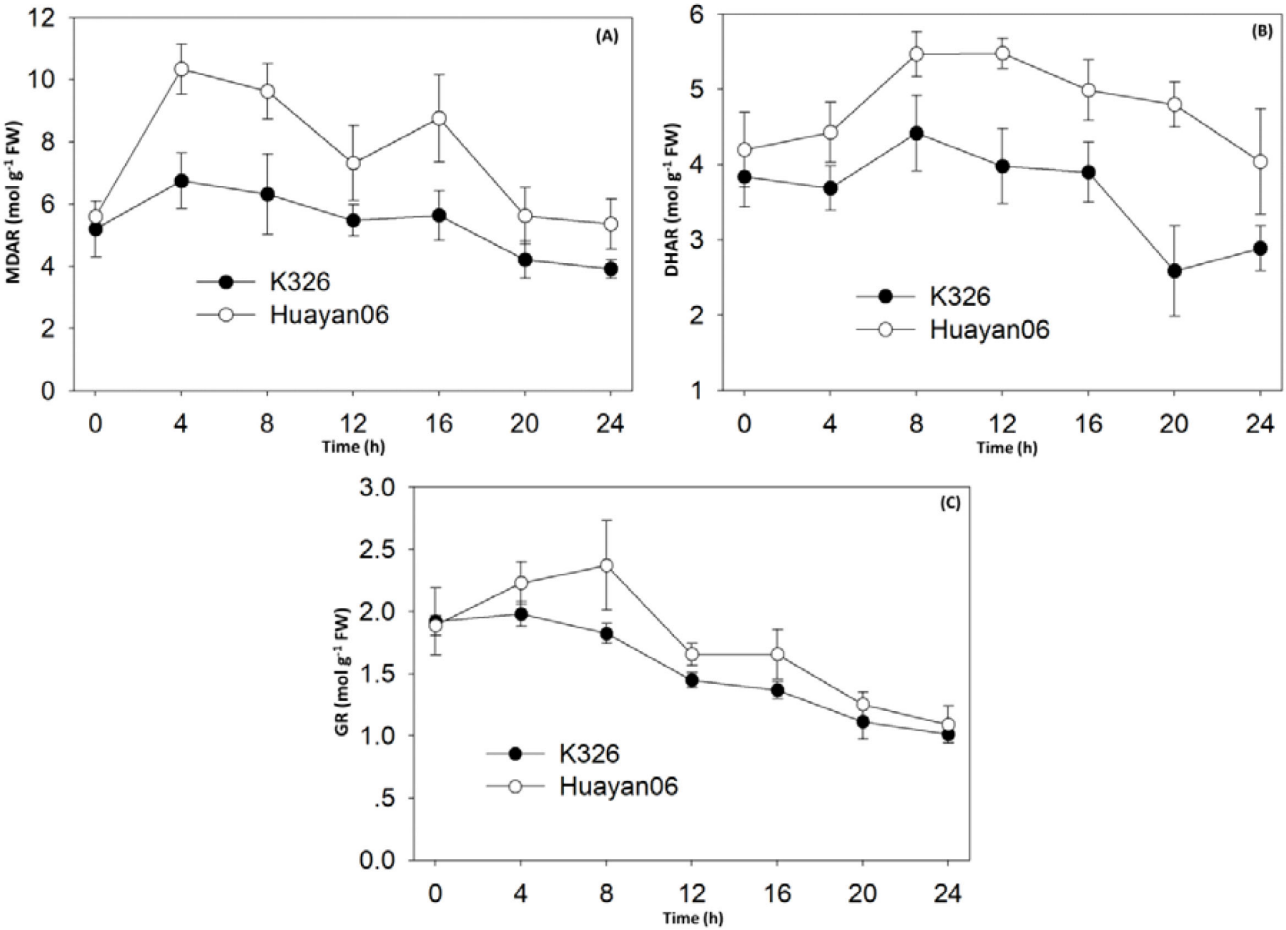
Effect of LT treatment time on activities of single DHA reductase **(A)**, DHA reductase **(B)** and GR **(C)** in flue-cured tobacco leaves.

**Figure 10** shows the changes in non-enzymatic antioxidant regeneration enzymes MDAR, DHAR, and GR activities in flue-cured tobacco leaves at different time periods under LT treatment. It can be seen from **Figures 10A, B** that the changes in MDAR and DHAR activities in different periods after LT treatment was consistent with the changes in ASA content. 0–4 h after LT treatment, the activities of MDAR and DHAR in leaves significantly increased, and the corresponding ASA content also increased considerably (see **Figure 9A**). Then, with the extension of treatment time, the activities of MDAR and DHAR gradually decreased, and the content of ASA in leaves also decreased. At the same time, it can be seen that the activities of MDAR and DHAR in Huayan 06 leaves were much greater than those in K326 during the entire treatment phase, which was consistent with the variation of ASA content among varieties under LT treatment. In addition, the response of GSH regenerating enzyme GR activity to LT stress (**Figure 10C**) was also consistent with the changing trend of GSH content (**Figures 9B, C**). However, during the treatment time of LT stress, the activities of MDAR and DHAR in the leaves of Huayan 06 were significantly higher than those of GR. The content of ASA was significantly higher than that of GSH, demonstrating that increasing the content of antioxidant ASA in tobacco plants under LT stress was an important reason for the strong resistance to LT of Huayan 06.

### 4.5 Effects of LT on photosynthetic metabolic enzymes and dry matter accumulation in Flue-cured tobacco

#### 4.5.1 Effect of LT on photosynthetic enzyme activity and PN of flue-cured tobacco leaves

**Figure 11:**
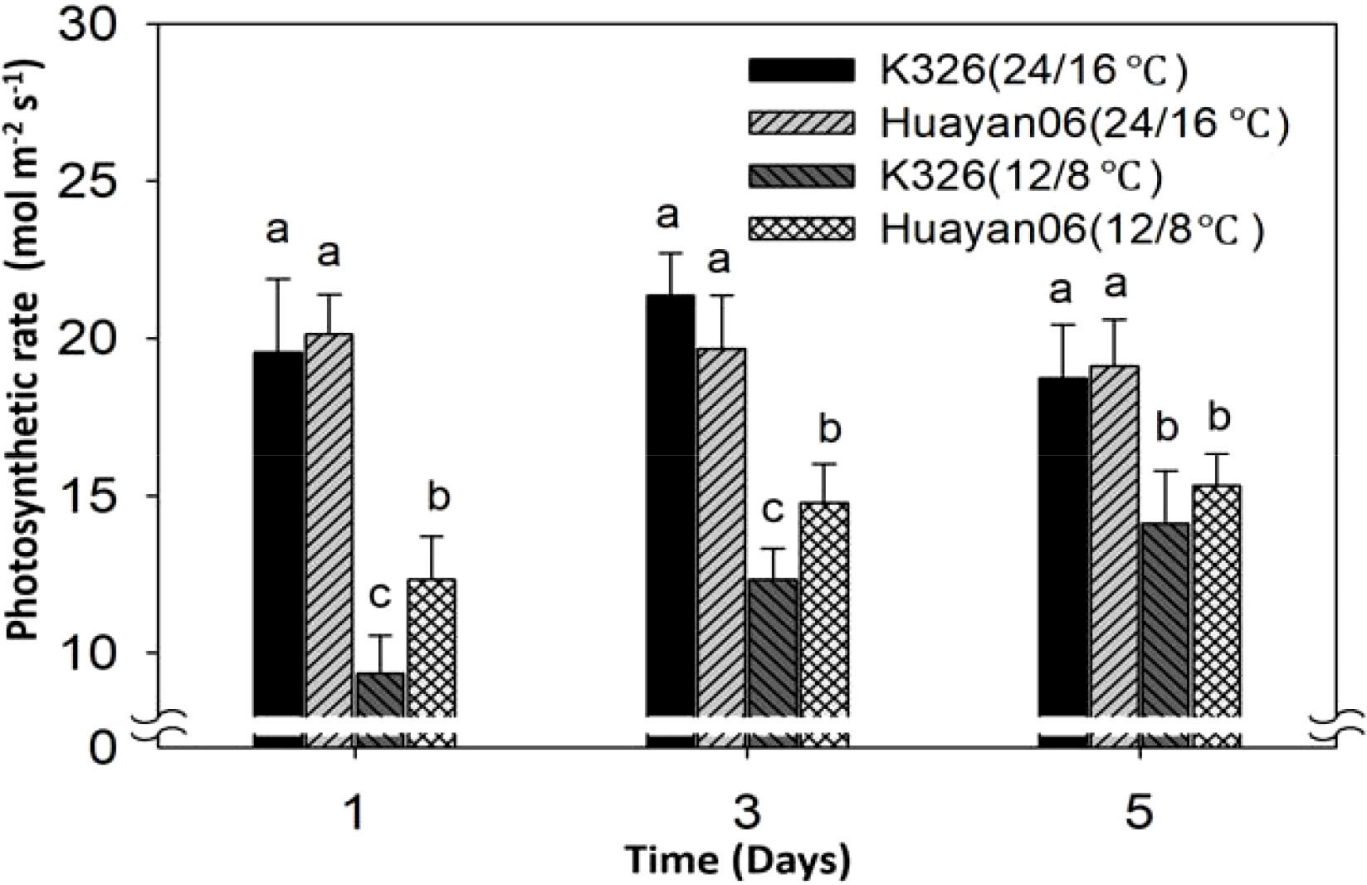
Effect of LT treatment time on PN of flue-cured tobacco leaves. Different letters in data bars represent significant differences (P ≤ 0.05).

**Figure 12:**
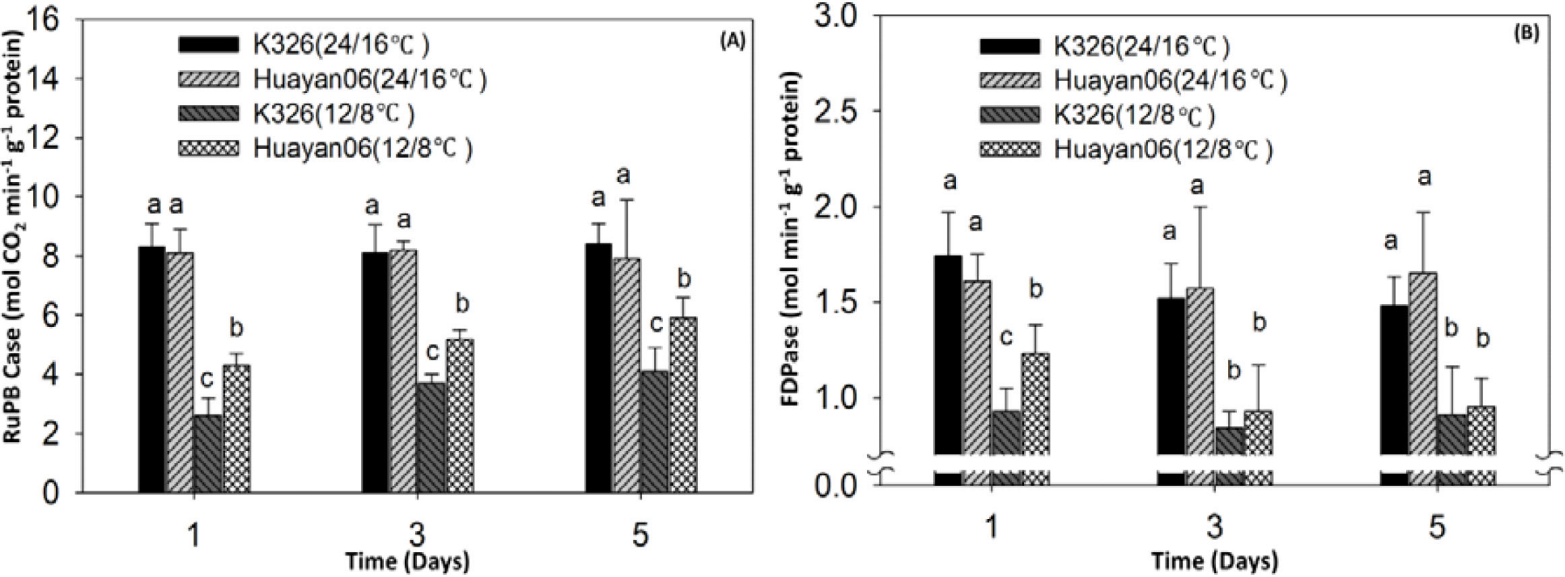
Effect of LT treatment duration on net PN rate in flue-cured tobacco leaves. Plants were grown under control conditions (24/16 °C) or low temperature stress (12/8 °C) for 1, 3, and 5 days. Different letters above the bars indicate statistically significant differences between treatments (P ≤ 0.05).

**Figure 11** indicates the differences in the activities of two key enzymes, ribulose-1,5-bisphosphate carboxylase (RuBPase) and fructose-1,6-bisphosphate esterase (FDPase), during photosynthesis of flue-cured tobacco leaves treated at different temperatures for different days. Under the test conditions, 24/16°C treatment for 1, 3, and 5 days had no considerable effect on the activities of RuBPase and FDPase. Accordingly, the PN of leaves had no significant change under this temperature treatment (see **Figure 12**), and there was no significant difference among varieties. However, LT treatment at 12/8°C significantly reduced the activities of RuBPase and FDPase (compared with 24/16°C). After LT treatment at 12/8°C for 1 day, the activities of RuBPase and FDPase in K326 flue-cured tobacco leaves were considerably lower than those in Huayan 06. Correspondingly, the PN of leaves also changed significantly, consistent with the changing trend of enzyme activity.

#### 4.5.2 Effect of LT on dry matter accumulation of Flue-cured tobacco

**Figure 13:**
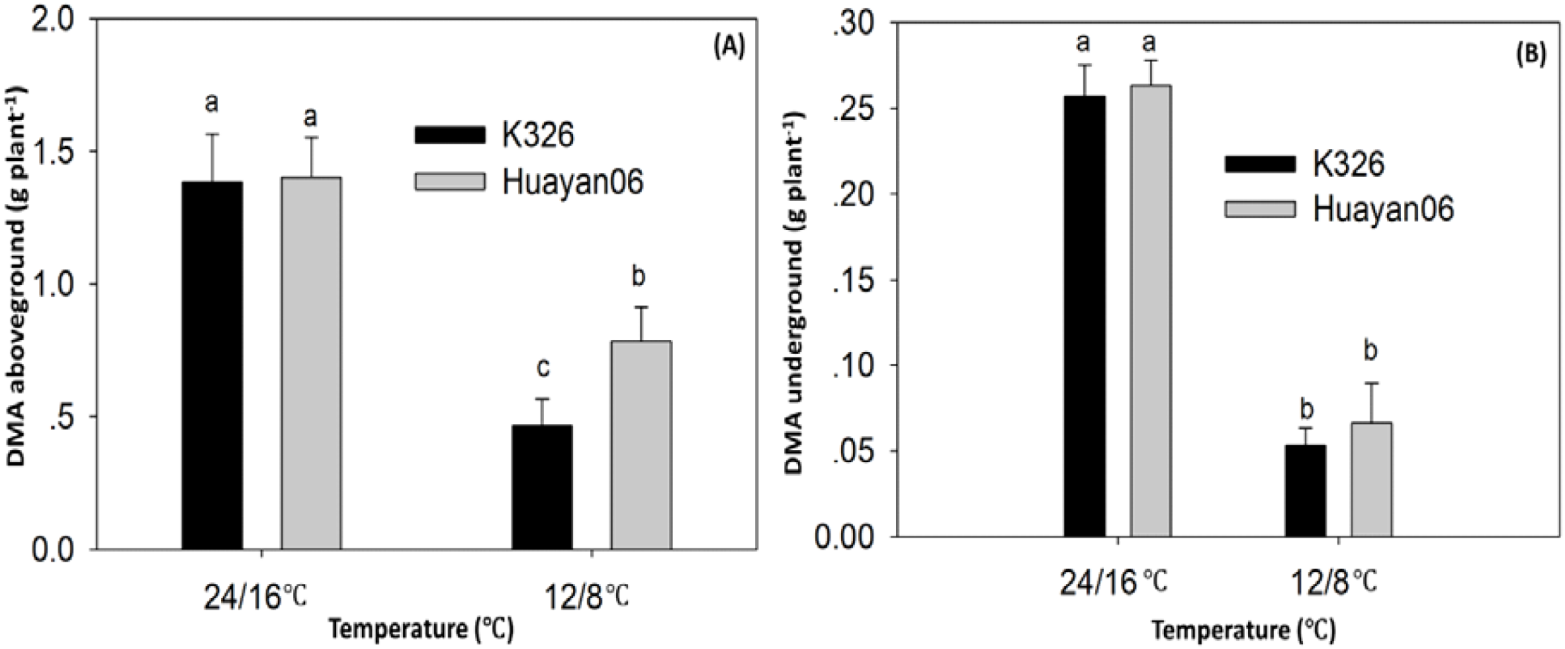
Effects of LT treatment on dry matter accumulation (DMA) aboveground **(A)** and underground **(B)** of flue-cured tobacco plants.. Different letters in data bars represent significant differences (P ≤ 0.05).

**Figure 13** shows the dry matter weight of aboveground (A) and underground (B) flue-cured tobacco plants after 15 days of treatment at different temperatures. There was no significant difference in the dry matter mass buildup of above-ground and subversive parts of the two varieties (lines) after 15 days of treatment at 24/16°C. Still, the dry matter accumulation of aboveground and underground parts of flue-cured tobacco after 15 days of treatment at 12/8°C was significantly reduced (compared with that at 24/16°C). There were differences among varieties in the dry matter accumulation of aboveground parts. Under 12/8°C LT treatment, K326 was more sensitive. Its dry matter accumulation was significantly lower than that of Huayan 06, decreasing 66.4%. In comparison, Huayan 06 decreased by 44.2%, indicating that under LT, the dry matter production capacity of Huayan 06 was significantly stronger than that of K326, showing a strong ability to resist LT.

## 5 Discussion

Temperature is one of the leading environmental factors affecting photosynthesis (Kumarathunge et al., 2019). Net PN is a sensitive index that reflects the operation of the photosynthetic mechanism. It constantly changes under the influence of various external environmental factors and plant internal factors (Song et al., 2016). The changes in net PN were affected by ecological and physiological factors, and there might be some interaction between these factors (Ahmad et al., 2022b). Prior experiments have demonstrated that the main factors causing the decrease in PN were the partial closure of stomata and the decline in photosynthetic activity of mesophyll cells (Sukhova et al., 2022). (Liu et al., 2020) believed that when Gs and Ci reduced simultaneously, the decrease in PN was mainly affected by stomatal limitation. If the increase of Ci accompanied the decrease of Pn, the main limiting factor of photosynthesis was the nonstomatal factor. In this study, with the decrease of treatment temperature, Pn and Gs decreased gradually. At the same time, Ci increased first (when the temperature decreased from 20°C to 12°C) and then reduced (when the temperature was lower than 12°C), indicating that in the process of temperature decreasing to mild LT (8°C), the limiting factor of photosynthesis was mainly nonstomatal factors. With the further decrease in temperature, nonstomatal and stomatal factors limited the photosynthesis of tobacco seedlings. However, nonstomatal factors were the main reason for the decrease in the net PN of tobacco seedlings affected by low night temperatures. In addition, the study also found that under LT treatment (6°C), within 8 h before LT treatment, the PN also decreased significantly with the extension of treatment time. After 12 h of LT treatment, the PN was zero. At this time, although the stomatal conductance was small, the intercellular CO_2_ concentration in leaves were still at a high level, which indicated that the photosynthetic activity of mesophyll cells was an important factor causing the decrease in PN after 12 h of LT treatment. Nonstomatal factors were the main influences leading to the decline of the photosynthetic rate, which was confirmed in this study. LT treatment significantly reduced the activities of two photosynthetic metabolic enzymes in leaves; At the same time, it was also found that nonstomatal factors and stomatal factors had a limiting effect on the PN with the further extension of LT treatment time. This is consistent with the research on LT control trees (Zhang et al., 2020) and Cucumbers (Shibuya et al., 2017). However, some studies have reported contradictory findings. The decrease of PN in coffee (Roonprapant et al., 2021) and Mango (Liu X. et al., 2022) caused by LT is mainly due to stomatal factors. This difference may be due to the different response mechanisms of photosynthesis of other plants to various environmental stresses or action modes and degrees and the diversity of responses to stomatal and nonstomatal factors (Jaffar et al., 2022). The comparison between the two varieties (lines) also found that the PN of flue-cured tobacco leaves decreased sharply with the LT treatment temperature. There were significant differences among varieties. Huayan 06 had better tolerance to LT in photosynthetic characteristics than K326, and the PN of Huayan 06 recovered significantly faster than K326 in the process of relieving LT stress, indicating that Huayan 06 had strong resistance to LT, it is of great significance for further discovery and utilization of cold resistant gene resources.

Chlorophyll fluorescence factors are a group of variables or constants used to define the mechanism of plant photosynthesis and photosynthetic physiological conditions, which reflect the characteristics of plant “internality”, and are regarded as internal probes to study the association between plant photosynthesis and the environment (Kalaji et al., 2018). Therefore, comparing with the “apparent” gas exchange parameters, analyzing the changes in chlorophyll fluorescence parameters helps determine the degree of photosynthetic mechanism affected. The maximum photochemical efficiency Fv/Fm of PSII is usually used to express the degree of photoinhibition, and the recovery ability and level of this ratio after removing stress can more accurately reflect the degree of photoinhibition damage and the ability to resist photoinhibition damage of plants (Tang et al., 2017). This analysis found that fv/fm did not change considerably when the treatment temperature was reduced from 24°C to 12°C. Still, with the further decrease in temperature, Fv/Fm decreased significantly. The reduction of K326 was more significant than that of Huayan 06, indicating that LT had a noticeable photoinhibition effect on the photosynthetic reaction center. In addition, this study also found that, with the extension of LT (6°C) stress time, the decrease of Fv/Fm in K326 was significantly greater than that in Huayan 06. In the recovery process of LT stress, the recovery rate of Fv/Fm in Huayan 06 was significantly faster than that in K326, indicating that the degree of light inhibition of Huayan 06 under LT stress was significantly less than that in K326. Φ_PSII_ is a measure of the capability of PSII to convert captured light energy into biochemical energy. It refers to the photochemical quantum efficiency of an open PSII reaction center with photochemical activity (Srivastava et al., 2009). The rise and fall of this value are closely related to the intensity of the carbon assimilation reaction. This study found that with the aggravation of LT stress and the extension of time, the more pronounced the downward trend of Φ_PSII_ is, and the decrease of Φ_PSII_ was significantly faster than F_v_’/F_m_’, and there was a significant difference among varieties (Chen et al., 2022). The decrease of Huayan 06 was significantly less than that of K326. QP reflects the opening degree of the PSII reaction center and is an assessment of the oxidation state of the primary electron acceptor of PSII. The decrease in its value indicates that the electron transfer of QA-QB is inhibited (He et al., 2021). F_v_’/F_m_’is the competition between the photochemical and heat dissipation processes in the PSII reaction center, so it is called to the proficiency of capturing excitation energy in the open PSII reaction center. The decrease indicates that a considerable part of the excitation energy captured by the blade has not been transferred to the PS II reaction center, which is likely to be dissipated from the capture antenna (Zivcak et al., 2013). In this study, the downregulation of PS II at LT was manifested by the decrease of QP and F_v_’/F_m_’; that is, the closing ratio of PS II reaction center increased, and the excitation energy capture efficiency decreased, and the decrease of K326 was significantly higher than that of Huayan 06. In conclusion, under LT stress, the degree of photoinhibition of Huayan 06 leaves was significantly less than that of K326. Similarly, the actual photochemical quantum efficiency and the efficiency of capturing excitation energy were significantly higher than that of K326, which may be one of the essential reasons why Huayan06 can still maintain a high PN under LT.

LT stress can increase the level of reactive oxygen species in cells and induce the establishment of a plant defense system to avoid or reduce the damage of reactive oxygen species to plants (Quan et al., 2008). SOD, POD, and CAT coordinate to form the protective enzyme system of plants, remove plants’ ROS, and improve plants’ adaptability to stress (Bhaduri and Fulekar, 2012). In the plant protective enzyme system, SOD is the “first line of defense” against the damage of reactive oxygen species (Lee et al., 2004). The results showed that LT (6°C) treatment significantly increased SOD activity within 8h, and there was no significant difference between the two varieties. Elevated SOD activity was also reported in other plants under LT stress, such as strawberries and cucumbers (Guerra et al., 2016; Siddiqui et al., 2022). (Nawaz and Bano, 2020) found that the SOD activity of cucumber leaves treated at 5°C decreased. In fact, this study also found that SOD activity decreased with the further extension of low-stress time, indicating that the intensity of LT stress and treatment time have different effects on SOD, which may be related to the adaptation mechanism of the whole antioxidant metabolic system. It was found that the activities of CAT and POD increased in the first 4 hours of LT stress treatment but inhibited the activities of CAT and POD in tobacco leaves. This is similar to previous studies on cowpea seedlings (Dadasoglu et al., 2022). However, it has also been reported that CAT activity in grafted cucumber leaves increased after LT stress (Jun-jie et al., 2009). It indicates that the different responses of antioxidant enzymes in various plant species may be due to their other tolerance mechanisms to stress and may also be related to plant species, size, multiple organs, and different LT stress degrees and times. The results of this study also confirmed this point. From the analysis of two varieties (lines), it was found that there was no significant difference in the activities of SOD, CAT, and POD in Huayan 06 and K326, but the activity of APX in Huayan 06 was significantly higher than that in K326. The activities of MDAR, DHAR, and GR, as well as the content of ASA and GSH in the leaves of Huayan 06, were significantly higher than that in K326 during the whole LT treatment time. The results showed that the high antioxidant capacity of Huayan 06 under LT stress mainly depended on the stimulation of the ASA defense system, which increased the activities of APX and DHAR for accelerating ASA recycling regeneration, thus alleviating the damage of LT to tobacco plants.

Tobacco is a thermophilic crop; if the temperature decreases, its growth will slow down rapidly (Ogbaga et al., 2018). Many studies have shown that LT significantly reduces plant height, the number of unfolded leaves, and leaf area (Ge et al., 2012; Jiang and Tian, 2010) and substantially reduces the fresh weight, dry weight, and root shoot ratio of roots, stems and leaves (Yang et al., 2018), indicating that when the environmental temperature is lower than the optimal temperature for plant growth and development, the growth and development of various organs of the plant will be adversely affected, or even irreversibly damaged (Castiglia et al., 2016). The dry matter accumulation of crops is the basis of crop growth and yield formation, and temperature is one of the leading environmental factors affecting the accumulation of dry matter, which can directly affect the photosynthetic capacity of crops and further affect the distribution of dry matter (Dai et al., 2015). It was found that 24/16°C treatment for 1, 3, and 5 days had no significant effect on the activities of RuBPase and FDPase. Accordingly, the PN of leaves had no significant change under this temperature treatment, and there was no significant difference among varieties (Vasiliadou and Dordas, 2009). However, LT treatment at 12/8°C decreased the activities of RuBPase and FDPase significantly (compared with 24/16°C). After 3 days of LT treatment at 12/8°C, there was no significant difference in FDPase activity between K326 flue-cured tobacco leaves and Huayan06, but the PN was significantly lower than Huayan 06.

From the dry matter accumulation and distribution perspective, the LT treatment at 12/8°C significantly reduced the dry matter accumulation of both aboveground and underground flue-cured tobacco after 15 days, with differences observed among varieties (Heuvelink and Dorais, 2005). Under the LT treatment at 12°C, K326 was more sensitive. Its aboveground dry matter accumulation was significantly lower than that of Huayan06, with a reduction of 66.4%. In comparison, Huayan06 exhibited a decrease of 44.2%, indicating that under LT stress, the dry matter production capacity of Huayan06 was significantly more substantial than that of K326, demonstrating strong LT resistance. These results indicated that Huayan06 had stronger resistance to LT than K326 under LT. It was mainly because Huayan06 stimulated the ASA defense system under LT stress, resulting in higher active oxidation capacity and less impact from LT stress. Consequently, Huayan06 maintained higher photosynthetic metabolic activity and better growth.

## 6 Conclusion

It has been determined that LT has a detrimental effect on tobacco crop growth and development and dramatically lower crop productivity. The present study reveals that stomatal and nonstomatal variables hampered the photosynthesis of tobacco seedlings as the temperature continued to drop. In addition, it was discovered that stomatal factors and nonstomatal variables had a controlling influence on the PN with the further extension of the LT treatment period. LT treatment considerably decreased the activities of two photosynthetic metabolic enzymes in leaves. When the treatment temperature was lowered from 24°C to 12°C, it was discovered that fv/fm did not change significantly. However, as the temperature was reduced further, Fv/Fm decreased significantly, and the decrease in K326 was more significant than that of Huayan 06, indicating that LT had a photo inhibitory effect on the photosynthetic reaction center. During the entire LT treatment period, Huayan 06 leaves considerably outperformed K326 in MDAR, DHAR, GR activities, ASA, and GSH levels. RuBPase and FDPase activity were unaffected by 24/16°C treatment for 1, 3, and 5 days. RuBPase and FDPase activity were dramatically reduced during LT treatment at 12/8°C compared to 24/16°C. Huayan 06 was more resistant to LT than K326 primarily because it increased the active oxidation capacity of the ASA defense system when exposed to LT.

## Data Availability

The original contributions presented in the study are included in the article/supplementary material. Further inquiries can be directed to the corresponding author/s.
